# A Proteomic Approach for Systematic Mapping of Substrates of Human Deubiquitinating Enzymes

**DOI:** 10.3390/ijms22094851

**Published:** 2021-05-03

**Authors:** Juanma Ramirez, Gorka Prieto, Anne Olazabal-Herrero, Eva Borràs, Elvira Fernandez-Vigo, Unai Alduntzin, Nerea Osinalde, Javier Beaskoetxea, Benoit Lectez, Kerman Aloria, Jose Antonio Rodriguez, Alberto Paradela, Eduard Sabidó, Javier Muñoz, Fernando Corrales, Jesus M. Arizmendi, Ugo Mayor

**Affiliations:** 1 Department of Biochemistry and Molecular Biology, Faculty of Science and Technology, University of the Basque Country (UPV/EHU), 48940 Leioa, Spain; juanmanuel.ramirez@ehu.eus (J.R.); unai.alduntzi@ehu.eus (U.A.); javier.beaskoetxea@ehu.eus (J.B.); benoitlouisphilippe.lectez@ehu.eus (B.L.); jm.arizmendi@ehu.eus (J.M.A.); 2 Department of Communications Engineering, University of the Basque Country (UPV/EHU), 48013 Bilbao, Spain; gorka.prieto@ehu.eus; 3 Department of Genetics, Physical Anthropology and Animal Physiology, University of the Basque Country (UPV/EHU), 48940 Leioa, Spain; ane.olazabal@ehu.eus (A.O.-H.); josean.rodriguez@ehu.es (J.A.R.); 4 Center for Genomic Regulation, Barcelona Institute of Science and Technology (BIST), 08003 Barcelona, Spain; eva.borras@upf.edu (E.B.); eduard.sabido@crg.cat (E.S.); 5 Department of Experimental and Health Sciences, Universitat Pompeu Fabra, 08003, Barcelona, Spain; 6 Proteomics Unit, Spanish National Cancer Research Center (CNIO), 28029 Madrid, Spain; efvigo@cnio.es (E.F.-V.); jmunozpe@cnio.es (J.M.); 7 Department of Biochemistry and Molecular Biology, Faculty of Pharmacy, University of Basque Country UPV/EHU, 01006 Vitoria-Gasteiz, Spain; nerea.osinalde@ehu.eus; 8 Proteomics Core Facility-SGIKER, University of the Basque Country (UPV/EHU), 48940 Leioa, Spain; kerman.aloria@ehu.eus; 9 Functional Proteomics Facility, Centro Nacional de Biotecnología (CNB-CSIC), ProteoRed-ISCIII, 28029 Madrid, Spain; alberto.paradela@cnb.csic.es (A.P.); fcorrales@cnb.csic.es (F.C.); 10 Ikerbasque, Basque Foundation for Science, 48013 Bilbao, Spain

**Keywords:** ubiquitination, deubiquitinating enzyme, quantitative proteomics, DUBase

## Abstract

The human genome contains nearly 100 deubiquitinating enzymes (DUBs) responsible for removing ubiquitin moieties from a large variety of substrates. Which DUBs are responsible for targeting which substrates remain mostly unknown. Here we implement the ^bio^Ub approach to identify DUB substrates in a systematic manner, combining gene silencing and proteomics analyses. Silencing of individual DUB enzymes is used to reduce their ubiquitin deconjugating activity, leading to an increase of the ubiquitination of their substrates, which can then be isolated and identified. We report here quantitative proteomic data of the putative substrates of 5 human DUBs. Furthermore, we have built a novel interactive database of DUB substrates to provide easy access to our data and collect DUB proteome data from other groups as a reference resource in the DUB substrates research field.

## 1. Introduction

Cells are very dynamic entities that are constantly synthesizing and degrading proteins in order to ensure their proper functioning. For protein removal, eukaryotic cells rely heavily on the Ubiquitin-Proteasome System (UPS), a quality control mechanism that targets dispensable, misfolded or proteins accumulated in excess for degradation. The proteins to be degraded are first covalently modified with K48- or K11-based polyubiquitin chains, built by the orchestrated activity of three enzymes, which are generically known as ubiquitin-activating E1, ubiquitin-conjugating E2 and ubiquitin-ligase E3. Ubiquitinated proteins are then directed to the proteasome, a multi-catalytic enzyme complex, where they are subjected to degradation [1]. However, this is not the only fate of ubiquitinated proteins [2]. When proteins are modified with ubiquitin chains (poly-ubiquitination) of different topologies, or when just a single ubiquitin (mono-ubiquitination) is attached at each ubiquitination site, this modification has different outcomes. As a result, in addition to proteasomal degradation [3], protein ubiquitination is also involved in a wide range of non-proteolytic biological processes, such as receptor endocytosis [4] or regulation of protein activity [5]. Similarly to other post-translational modifications, the UPS pathway also has a mechanism to reverse the effect of E1, E2 and E3 enzymes through the activity of a fourth family of enzymes, the deubiquitinating (DUB) enzymes [6]. DUBs are encoded by ~100 genes in humans that, based on sequence and structural similarities of their catalytic domain, are classified into seven families: Ubiquitin-specific proteases (USPs), Ubiquitin *C*-terminal Hydrolases (UCHs), Ovarian Tumour proteases (OTUs), Machado-Joseph disease proteases or Josephins (MJDs), MIU-containing novel DUBs (MINDYs), Zinc finger containing Ubiquitin Peptidase 1 (ZUP1) and JAB1/MPN/MOV34 (JAMMs) proteases [6]. The major roles of DUBs can be summarized as; (i) regulation of free ubiquitin levels [7]; (ii) prevention of premature degradation of substrates by rescuing them from the proteasome [8]; (iii) contribution to proteasomal degradation facilitating the entry of substrates to the proteasome [9]; and (iv) dynamic regulation of protein activity [10,11]. Similarly to E3s [12,13], DUB enzymes show substrate specificity, and furthermore, particular DUBs may also exhibit a preference for certain ubiquitin chain linkages [6]. Therefore, our hypothesis is that each DUB will have its own repertoire of target substrates on specific cell types.

Failures in the Ubiquitin Proteasome System lead to the development of a number of disorders, such as Parkinson’s disease [14], cancer [15], Angelman syndrome [16] or numerous rare neurological diseases [17]. Hence, identification of the ubiquitome (the complement of ubiquitinated proteins) under physiological and disturbed conditions can provide a better understanding of the role of ubiquitination. Eventually, this might contribute to the development of novel therapeutic strategies for diseases where the UPS is altered. DUBs are the most likely enzymes on the UPS pathway to be used as therapeutic targets, given the ease by which they can be inhibited, as well as due to their relatively defined specificity as compared for example to the proteasome. Therefore, interest in the pathways they regulate is rapidly growing [18]. Obviously, in order to better understand how they could be used for therapeutic purposes, identifying the protein substrates of each DUB would represent an important step forward.

For any given protein, only a small percentage of the molecules are usually ubiquitinated at a given time and tissue. The substoichiometric nature of ubiquitination poses a challenge in the study of this highly labile post-translational modification. Different strategies have been developed for the enrichment of ubiquinitated proteins. One of the most popular strategies is based on the use of antibodies recognizing the diGly signature that the ubiquitinated peptides retain after tryptic digestion [19,20]. However, as enrichment is performed at peptide level, this approach precludes further work, such as Western blot validation, to be done at the protein level. Another popular tool to identify ubiquitinated proteins are the TUBEs, tandem ubiquitin binding entities that can capture ubiquitinated material from different types of samples [21]. In this case, the main problem is that due to the use of non-denaturing buffers during the purification protocol, a plethora of proteins associated to the ubiquitinated material are also isolated. To overcome the limitations of both strategies, we developed the ^bio^Ub strategy that can be used for the efficient isolation of ubiquitin conjugates from flies, mice and human cell lines [22,23,24,25,26,27,28,29,30]. This strategy is based on the in vivo biotinylation of ubiquitin (thus ^bio^Ub strategy), which, due to the strength and the specificity of the avidin-biotin interaction, allows the isolation and enrichment of ubiquitinated proteins under extremely stringent conditions [22]. The ubiquitination levels of cellular proteins can be quantitatively monitored with high reproducibility combining the ^bio^Ub strategy and MS analysis (Figure S1A). This approach had been successfully used to detect the increased ubiquitination of E3 ligase substrates upon the overexpression of E3s, such as Parkin, Ube3a and Ariadne-1 [29,30,31]. DUB enzymes remove ubiquitin moieties from their substrates, providing the cell with a control mechanism for the ubiquitinating action of E3 ligases. Due to the low intrinsic level of basal ubiquitination, the most efficient approach to identify the substrates of any given DUB by proteomics is to inhibit or silence it [32]. The silencing of a particular DUB renders a higher ubiquitination of its substrates, relative to a control sample (Figure S1B). Hence, by comparing proteins present in the ^bio^Ub enriched fraction of a control and a DUB-silenced sample, it is possible to identify those proteins whose ubiquitination level has increased upon DUB silencing, and can be therefore considered as putative substrates of that enzyme.

In the present work, we have applied this strategy to identify substrates of 5 members of the USP family of DUB enzymes (USP1, USP7, USP9X, USP11 and USP42). After silencing each of the 5 DUBs, ubiquitinated proteins have been enriched using the ^bio^Ub strategy, and subjected to label-free quantitative proteomic analysis. Additionally, the lack of available online resources for substrates of DUB enzymes has prompted us to build an interactive web-based database (https://ehubio.ehu.eus/dubase, accessed date 30 April 2021), named DUBase (DUB database), in order to provide easy access to our data and with the scope to integrate data from other groups in the future.

## 2. Results and Discussion

### 2.1. Identification of DUB Substrates in Cell Culture

The ^bio^Ub system has been here applied to human cells in culture in combination with an RNA silencing protocol. Cells are first transfected with a siRNA to silence a certain DUB, and then, supplied with a biotinylated ubiquitin by transfection of the ^bio^Ub construct [22], a precursor polypeptide composed of six biotinylatable versions of ubiquitin and the biotin ligase enzyme of *E. coli*, BirA (Figure S1A). Once this precursor is digested by endogenous DUBs and ubiquitins and BirA are released, the BirA enzyme is able to label the 16 amino acid long motif that each ubiquitin bears at their *N*-terminal part with a biotin molecule. This small biotinylatable tag has been described to be the minimal peptide that BirA can efficiently biotinylate [33], with very minor off-targets [22,25,26,27,30]. After a 4-day protocol, HEK293 cells are ready for the isolation of the ubiquitinated material, which can be either analysed by mass spectrometry or Western blot (Figure S1C).

**Figure 1:**
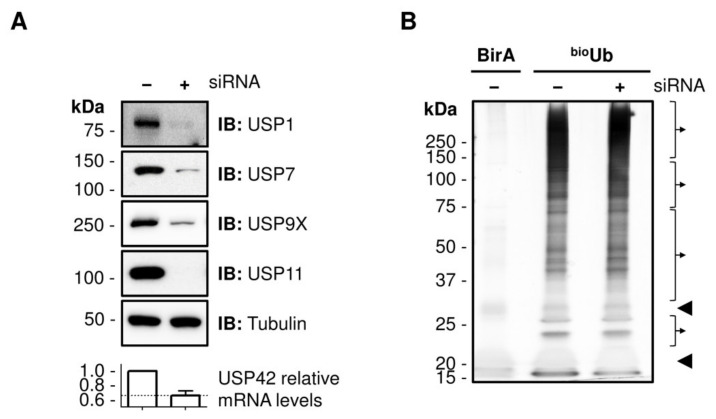
Isolation of ubiquitinated material by biotin pull-downs in siRNA silenced samples. (**A**) Analysed deubiquitinating enzymes (DUBs). The reduction levels achieved for each of the DUB analysed are shown. USP1, USP7, USP9X and USP11 levels were detected by Western blot, whereas RT-qPCR was used for USP42. The mRNA levels were normalized to the control samples. The average mRNA level in USP42-silenced sample (0.65) is shown with a dashed line. (**B**) Silver staining of the material purified by biotin-pulldowns. Just a few endogenous carboxylases proteins are detected in samples overexpressing only the BirA enzyme (BirA sample). However, a smear corresponding to ubiquitinated material is detected when the BirA enzyme and the biotinylated ubiquitins are co-expressed together (^bio^Ub samples). Similar amount of ubiquitinated material is isolated from cell treated with the siRNA control (−) and a DUB-specific siRNA (+). Avidin monomers and dimers are indicated with arrowheads. The four slices that are extracted from each sample for MS analysis are highlighted with the brackets. Only the ^bio^Ub samples were analysed using the LFQ MS approach.

We silenced independently five DUBs from the USP family: USP1, USP7, USP9X, USP11 and UPS42, as confirmed by Western blotting or in the case of USP42, due to lack of suitable antibodies, by quantitative RT-PCR (Figure 1A). Silencing of these five DUBs had no effect in the global levels of conjugated ubiquitin (Figure S2), and the amount of ubiquitinated proteins isolated from the cell extracts by biotin pull-downs was equivalent in both the ^bio^Ub control and DUB-silenced samples (Figure 1B and Figure S3).

**Figure 2:**
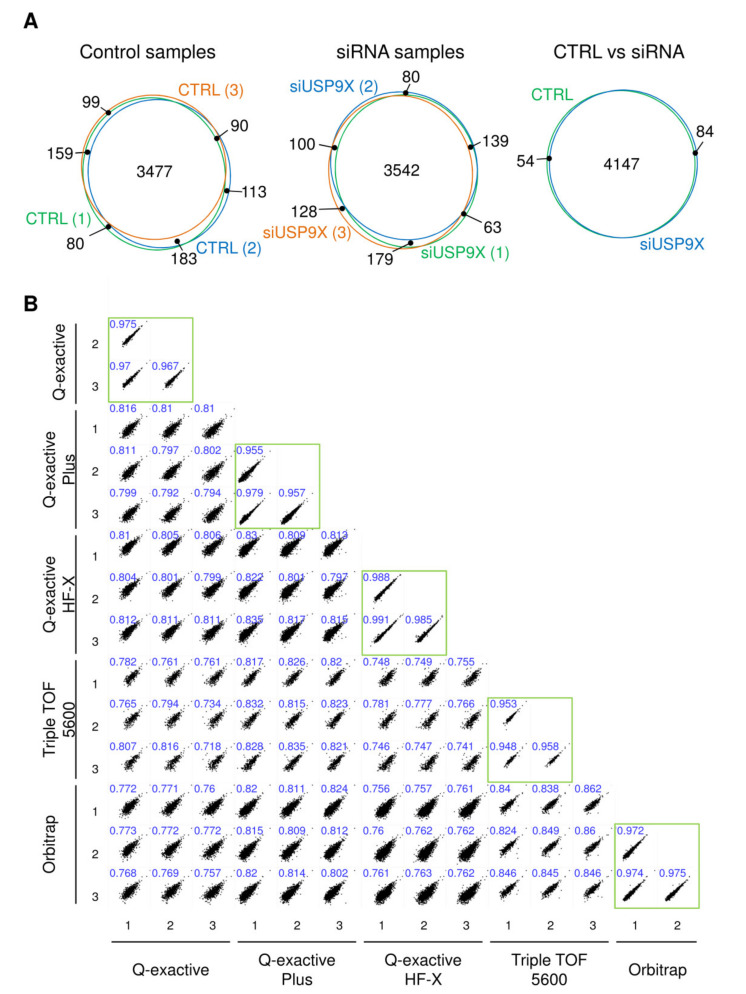
Reproducibility of the mass spectrometry analyses. (**A**) Overlap between proteins identified in control and siUSP9X samples. Venn diagrams showing the overlap of the proteins identified among the three replicas of each condition, as well as between both conditions are shown. (**B**) Multicorrelation graphs of label free quantification (LFQ) intensities (in log_2_ scale) of proteins identified across the control replicas of all the experiments. Pearson’s correlation coefficient is shown in blue. Correlations of the three replicas of the same experiment are highlighted with a green square.

**Figure 3:**
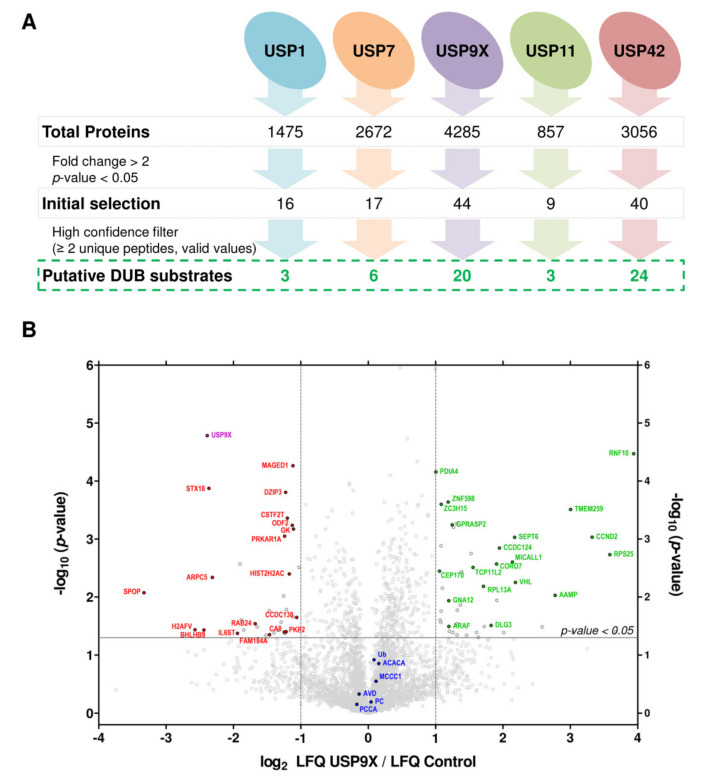
Identification of putative deubiquitinatint enzyme (DUB) substrates. (**A**) Summary of the putative substrates identified for each DUB. Proteins with a significant 2-fold increase of their abundance upon DUB-silencing were considered to be more ubiquitinated (initial selection). Of those, only proteins with at least two unique peptides and with low levels of imputation during the statistical analysis were considered as putative DUB substrates. (**B**) Putative substrates of the USP9X DUB. Volcano plot shows differentially ubiquitinated proteins upon USP9X silencing, relative to control samples. Abundance of each individual protein was determined by their label free quantification (LFQ) intensities. The LFQ siRNA/control ratios (log_2_ scale) and the *t*-test *p*-values (−log_10_ scale) are displayed in the *X* and *Y* axis, respectively. The threshold for statistical significance (*p*-value < 0.05) is indicated with a horizontal grey line, while vertical grey lines depict a 2-fold increase or decrease of the ubiquitinated levels upon DUB silencing. Proteins with a 2-fold increase or reduction of their ubiquitination levels upon USP9X silencing are shown in green and red respectively. USP9X is shown in purple. Endogenously ubiquitinated proteins (ACACA, MCCC1, PC and PCCA), ubiquitin (Ub) and avidin (AVD) are coloured in blue.

To assess the universality and robustness of this approach, we aimed to detect in vivo substrates of USP1, USP7, USP9X, USP11 and USP42 by performing triplicate proteomics experiments for each of the DUBs, in five different instruments from four separate laboratories. The control silencing sample was equivalent for all five experiments, and ^bio^Ub samples from control and DUB-silenced cells were analysed by label-free quantitative proteomics. A total of 5810 proteins were identified across the five experiments (Table S1). A high reproducibility across biological replicas of the samples was observed, as illustrated by the Venn diagrams for the USP9X data (Figure 2A). Overlap between the aggregated values of the control samples and the silenced samples was also high, in the order of 80–95% (see also Figure S4A). Comparison of control samples in all instruments indicates that, although the coverage depth varies across the different instruments due to their different technical capabilities (Figure 3A and Figure S4A), as much as 70% of the total proteins identified were detected in at least two out of the five experiments (Figure S4B). Moreover, when control experiments carried out in different labs were compared in pairs, more than 80% of the proteins from the smaller dataset were always found in the larger dataset, reflecting a high overlap between analyses (Figure S4C). A high reproducibility of the protein abundance was also observed across control sample replicas, both within each instrument and across different laboratories (Figure 2B). As anticipated, proteins consistently quantified across the multiple replicas within a given instrument or across different instruments were those presenting a higher abundance, as determined by their LFQ Intensity values (Figure S4D,E). Altogether, data showed that our approach can be easily applied in any laboratory providing highly reproducible results.

A total of 1277 GlyGly peptides, corresponding to 635 proteins, were identified across the different data sets; similarly, to the protein identifications, the highest coverage was observed for the USP9X dataset (Table S2).

### 2.2. Large Scale Detection of In Vivo Substrates for USP1, USP7, USP9X, USP11 and USP42

To our knowledge, this is the first large scale proteomic analysis focused on USP1, USP7, USP9X, USP11 and USP42 DUB enzymes. The total number of quantified proteins in each experiment varied significantly, most probably due to the different capabilities of each MS instrument. For example, in the USP11 and USP9X silencing experiments 857 and 4285 ubiquitinated proteins were quantified, respectively (Figure 3A). Our quality control standards indicated that the amount of biological material (as quantified using endogenously biotinylated proteins, such as ACACA, HLCS, MCCC1, PC and PCCA), and the total ubiquitinated material (as quantified using both endogenous and ectopic ubiquitin) were in general not altered between control and DUB silenced samples (Figure 3B and Figure S5). The exception was a small reduction of the ubiquitin levels observed when USP42 was silenced (Figure S5). Given the fact that USP42 silencing actually induces significant changes on the ubiquitination levels of 10 different DUBs (BAP1, JOSD1, OTUD5, SHMT1, UFD1L, USP9X, USP11, USP13, USP24, USP25) and at least 13 different E3s (Table S1), it could be hypothesized that the changes on ubiquitin levels are a result of a complex systemic change of ubiquitination dynamics.

The enrichment of ubiquitinated proteins upon DUB silencing was determined by Student’s *t*-test (Figure 3B and Figure S5). Proteins that showed a significant 2-fold increase of their LFQ intensity relative to control samples (siRNA LFQ/Control LFQ > 2, *p*-value < 0.05) were considered to be more ubiquitinated in DUB-silenced samples. Further, only proteins that fulfilled additional stringent criteria (see Data analysis section) were classified as putative DUB substrates. Hence, among all the proteins that were detected as more ubiquitinated in DUB-silenced samples (16 for USP1; 16 for USP7; 44 for USP9X; 9 for USP11; and 39 for UPS42), a minor fraction were considered as putative substrates (3 for USP1; 5 of USP7; 20 for USP9X; 3 for USP11; and 23 for UPS42) (Figure 3A and Table 1). As an example of the validity of the strategy developed here, we detected PCNA as a substrate of USP1 (Table 1 and Figure S5), in agreement with earlier reports [34]. Upon analysis of the isolated ubiquitinated material by immunoblotting, we further confirmed PCNA to be more ubiquitinated when USP1 is silenced (Figure S6A), underlining the capacity of this strategy to detect both by MS and by Western blotting the substrates of the investigated DUBs. We found barely no overlap between the putative substrates of the investigated five DUBs (Figure S6B), underlying that DUBs are highly specific enzymes [35]. The E3 ubiquitin-protein ligase RING2 (RNF2) is the only protein that appeared to be substrate of more than one DUB (Table 1). Altogether, these results illustrate the feasibility of the strategy followed in this study to uncover in vivo DUB substrates, providing also the opportunity to biochemically validate them by immunoblotting using the same sample. The Western blot analysis allows to elucidate whether a given substrate is mono or polyubiquitinated (Figure S6A).

As can be observed from the various volcano plots, in addition to identify many enriched ubiquitinated proteins, we also detected a number of proteins that were less ubiquitinated upon DUB silencing (labelled in red in Figure 3B and Figure S5). Most of those are likely to be downstream secondary effects of the ubiquitination changes observed in the right quadrants of the volcano plots. But, additionally, amongst those proteins on the left quadrant, we found USP1, USP7 and USP9X on their respective experiments (labelled in purple in Figure 3B and Figure S5). Their presence in the dataset indicates that those DUBs are themselves ubiquitinated under physiological conditions. Once they are silenced their ubiquitinated amount going down is therefore the expected consequence of their overall levels being reduced (or eliminated) due to the RNAi silencing.

From a biological perspective, our unbiased approach can greatly contribute to the identification of until now unknown pathways regulated by the different DUBs. Some of the substrates identified in our analysis (Table 1) are linked to already known functions of the studied DUBs. There are multiple examples of this within the DUB-specific substrates identified in this work. One of those is PCNA, which is involved in the USP1 dependent DNA repair process [36]. TTK protein kinase that is associated with cell proliferation [37], a process that has been reported to be regulated by USP7 [38]. RNF10 E3 ligase whose rat homolog is important for myelin formation [39], thus possibly contributing to the neuronal pathogenesis caused by USP9X mutations [40]. The ubiquitin conjugating UBE2L3 enzyme, reported to be involved in the regulation of cell cycle [41], in line with similar observations for USP11 [42]. And the WARS Tryptophan—tRNA ligase that has been associated with angiogenesis [43], a critical process in tumorigenesis, with which USP42 has also been linked [44]. Nevertheless, other substrates are linked to other cellular pathways, which indicate that the characterized DUBs will also regulate further pathways that have not yet been described. These new pieces of information will therefore be a high valuable resource for other labs working with DUB enzymes, as they might help to open new research field directions for these DUBs.

### 2.3. DUBase: A Novel Database for DUB Substrates

Given the current absence of any available deubiquitinating enzyme (DUB) substrate database, we considered that creating an open and flexible repository would provide a very valuable tool to the scientific community. We have therefore built DUBase, a freely accessible interactive database, containing the proteomic data for -so far- the 5 DUBs described in this paper. This database will be updated by the authors of this paper both by importing the results from new DUB substrates publications and upon request of members of the scientific community. DUBase can be publicly accessed at https://ehubio.ehu.eus/dubase.

**Figure 4:**
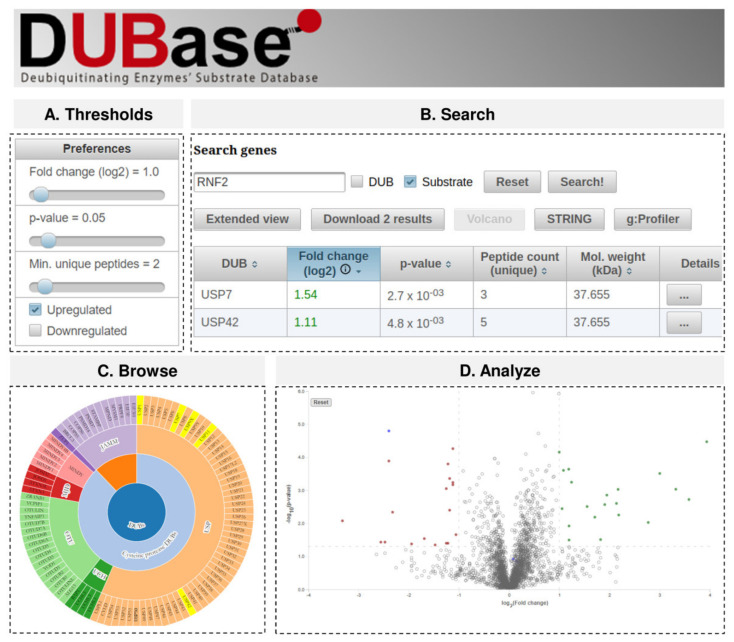
Deubiquitinating enzymes’ (DUBs) substrates database (DUBase). Example of the different tabs available in the web site are depicted. (**A**) Default threshold values can be customized to meet the user preferences, and these settings will be considered by the rest of the tabs during the user session. (**B**) The search tab allows querying by a given DUB name or by its substrate name and displays a table with the results. Then, the user can navigate the information in the database by clicking the different results, download them in CSV file format, display an interactive volcano plot (which also shows the identifications below the thresholds), and also can communicate the results to other web tools such as STRING or g:Profiler to identify affected pathways. (**C**) A simple alternative to reach these results for a given DUB is to use the browse tab, which displays in an interactive hierarchical chart the different DUB classes and highlights those for which there is information in the database. (**D**) In the analyse section gene ontology searches or volcano plots can be performed.

Proteomic data can be analysed using different criteria. For this reason, we have developed this database with an open option to redefine the thresholds that the user can adjust in terms of how a protein is defined as a putative DUB substrate (Figure 4A). We believe that a 100% increase on the amount of the ubiquitinated protein (as determined by LFQ values) is required for a DUB substrate to be considered as such (Fold change siDUB/ctr ≥ 2; or in log_2_ scale ≥ 1). But for other quantification protocols (e.g., TMT) a different threshold might be more appropriate. Additionally, some users might view our LFQ criteria as too conservative and might consider that a 20% increase on the amount of ubiquitinated protein is sufficient for a DUB substrate to be considered as such. An option to adjust both the fold-change and *p*-value thresholds, as well as the minimum number of unique peptides, is therefore provided in order to apply these criteria to any further analysis of the data on the repository. Additionally, the option to search for those proteins whose ubiquitination is reduced is also given, despite the reduction in ubiquitination levels from our experimental set up is more difficult to interpret. In this case, a Fold change ≥ 2 would provide a list of proteins whose ubiquitination levels have been reduced at least to the half. Once the user’s preferences are saved, they will be active during the analysis until the researcher defines new thresholds.

User may be interested in getting a list of substrates regulated by the DUB they are working with. Alternatively, they may want to check if a specific protein is regulated by any DUB. Consequently, DUBase provides two search options: (a) to query for the substrates of one DUB and (b) to check whether a specific protein is regulated by any DUB (Figure 4B). To search for DUB substrates, the user must type the name of the DUB in the search option and select the DUB box. Alternatively, the user may navigate throw the entire DUB family in the browse option and select the interested DUB (Figure 4C). These two options will provide a list of proteins that upon silencing of the selected DUB fulfill the previously established thresholds. For instance, when looking for USP7 substrates, the database will display a list of 6 putative candidates (log_2_ fold change siDUB/Ctr > 1, *p*-value < 0.05 and minimun 2 unique peptides) (Table 1). On the contrary, by typing the name of any given protein in the search option and selecting the substrate box, the database will provide the names of the DUBs that may deubiquitinate that given substrate. For example, if RNF2 gene name is typed in the search option, two DUBs will be displayed, USP7 and USP42. In relation to the experiments performed with each DUB, information regarding fold changes, *p*-values, unique peptide counts and molecular weight of the substrate will be shown at first in a tabulated form (Figure 4B). However, there is an option to view more information (protein ID, all peptide counts, sequence coverage and GlyGly site positions) by clicking the “extended view” button. Moreover, by clicking on the details (last column), a summary table is generated. In addition to the experimental data shown in the main interface, the table allows checking the methodological details (cell, organism, method, experimental conditions and supporting files) of the experiment. Furthermore, by clicking on gene and protein ID, there is direct access to Uniprot database. And in order to check whether the selected protein of interest is modified, there is also direct access to the PhosphoSite Plus [45] database.

In addition to retrieve experimental data and redirect to distinct databases, DUBase can be used to perform a number of analyses. Under the “Analyze” option, protein subsets from the database can be selected in order to be analyzed by String [46] or g: Profiler [47] to perform protein interaction and gene ontology analyses, respectively. The user can also build Volcano plots (Figure 4D). The thresholds previously defined (fold change and *p*-value) will be shown directly in the plot, and significantly regulated proteins will be coloured either in green (up-regulated) or red (down-regulated), whereas endogenously ubiquitinated proteins will be coloured in blue. By placing the cursor in any spot of the plot, gene name, description, fold change and *p*-value corresponding to such protein will also be shown.

## 3. Conclusions

Here we uncover the substrates of five different DUB enzymes with an easy-to-perform and well-established strategy, which can be implemented in other laboratories for the identification of further DUB-regulated proteomes. The application of such a strategy in multiple laboratories in parallel has a strong potential to fully map the complete landscape of DUB substrates across different cell lines, tissues and physiological and pathological conditions. Additionally, we have created a novel interactive database of DUB substrates that collects DUB proteome data as a central proteomics resource for the identification of DUB substrates. Given the growing interest on the inhibition of DUB enzymes as a potential therapeutic venue, describing the pathways regulated by those becomes ever more important.

## 4. Materials and Methods

### 4.1. Cell Culture and RNA Silencing

HEK293 cells were cultured under standard conditions (37 °C, 5% CO_2_) in Dulbecco’s modified Eagle medium/nutrient mixture F-12 (DMEM/F-12) with GlutaMAX (Gibco, Gaithersburg, MD, USA), supplemented with 10% fetal bovine serum (Gibco, Gaithersburg, MD, USA), 100 U/mL of penicillin (Gibco, Gaithersburg, MD, USA) and 100 µg/mL of streptomycin (Gibco, Gaithersburg, MD, USA). A total of 13.5 × 106 cells were seeded in 150 mm dishes for proteomics experiments. The following day, the medium was replaced by new one, but without any antibiotics. Then, siRNA treatments were performed using Lipofectamine RNAiMAX kit (Invitrogen, Carlsbad, CA, USA) according to the manufacturer’s instructions. Two different siRNAs (10 nM each) were used at the same time for the silencing of each of the DUB analysed, whereas a scramble siRNA (10 nM) was used as control. The following Silencer Select Validated siRNAs (Ambion, Austin, TX, USA) were used for the silencing of each of the human DUB: s14724 and s14725 for USP1; s15439 and s15440 for USP7; s15742 and s15743 for USP9X; s15739 and s15740 for USP11 and s38540 and s38541 for USP42. A Silencer Select siRNA (Ambion, Austin, TX, USA) with no significant sequence similarity to mouse, rat or human genes sequences was used as negative control (catalogue#4390843). All siRNA were used at a final concentration of 10 nM. Next day, i.e., 48 h after seeding, transfection of the ^bio^Ub plasmid was carried out with Lipofectamine 3000 reagent (Invitrogen) according to manufacturer’s instructions. Additionally, the medium was supplemented with 50 µM biotin solution. Cells were harvested 24 h later, i.e., 72 h after seeding, and pellets frozen until being used for Western Blot or biotin pull-downs (Figure S1C).

### 4.2. Real-Time Quantitative PCR

Total RNA was isolated from 1.2 × 106 HEK293 cells transfected with control siRNA or USP42 siRNA. RNA was extracted and further purified using the RNeasy Mini Kit (QIAGEN, Hilden, Germany) according to the manufacturer’s protocol. Contaminating genomic DNA was removed by treatment with deoxyribonuclease I (QIAGEN, Hilden, Germany), and cDNAs were synthesized from 1 μg of RNA using the AffinityScript Multi Temperature cDNA Synthesis Kit (Agilent Technologies, Santa Clara, CA, USA). Quantitative RT-PCR was performed on cDNA, in the presence of Power SYBR Green PCR Master Mix (Applied Biosystems, Foster City, CA, USA) containing preset concentrations of deoxynucleotide triphosphates and with specific primers, using the ABI Prism 7900 Sequence Detection System (Applied Biosystems, Foster City, CA, USA). PCR parameters were 50 °C for 2 min, 95 °C for 10 min, 40 cycles at 95 °C for 15 s and 60 °C for 1 min. The purity of the PCR products was assessed by dissociation curves. The amount of target cDNA was calculated by the comparative threshold (Ct) method and expressed by the 2^−ΔΔCt^ method, according to Applied Biosystems’ instructions, using glyceraldehyde-3-phosphate dehydrogenase (GAPDH) as an internal control. Expression of GAPDH mRNA was not affected by siRNA transfection, and the ratio of ΔCt value did not vary with the amount of cDNA. Each primer set was used at its optimal concentration (300 nM) with maximal efficacy. It was verified that one single specific product was amplified as shown by analysis of its melting temperature value.

Primers GAPDH:

Forward: 5-TGTGGGCATCAATGGATTTGG-3′
Reverse: 5-ACACCATGTATTCCGGGTCAAT-3′

Primers USP42:

Forward: 5-AATCTTCAGACCCATCAGCCT-3′
Reverse: 5-AGAACCTGCATCCATGTCTCC-3′

### 4.3. Western Blot

Proteins were resolved by SDS-PAGE in 4–12% Bolt Bis-Tris Plus pre-cast gels (Invitrogen, Carlsbad, CA, USA) and then transferred to PVDF membranes using the iBlot system (Invitrogen). Membranes were developed using the Clarity Western ECL substrate kit (Bio-Rad, Hercules, CA, USA) and the ChemiDocTM MP imaging system (Bio-Rad, Hercules, CA, USA). The following primary antibodies were used: goat anti biotin-horseradish peroxidase (HRP)-conjugated antibody (Cell Signaling Technology, Danvers, MA, USA; catalogue number 7075) at 1:1000; USP1 (Cell Signaling Technology, Danvers, MA, USA; catalogue number 8033); rabbit polyclonal anti-USP7 antibody (Bethyl laboratories, Montgomery, TX, USA; catalogue number A300-033A) at 1:3000; rabbit polyclonal anti-USP9X antibody (ProteinTech, Rosemont, IL, USA; catalogue number 55054-1-AP) at 1:1000; rabbit monoclonal anti-USP11 (Abcam, Cambridge, UK; catalogue number ab109232); mouse monoclonal anti-Tubulin beta antibody (Developmental Studies Hybridoma Bank, Iowa City, IA, USA; catalogue number E7) at 1:1000). The following secondary antibodies were used: goat anti-mouse-HRP-labelled antibody (Invitrogen, Carlsbad, CA, USA; catalogue number 62-6520) at 1:4000; goat anti-rabbit-HRP labelled antibody (Cell Signaling Technology, Danvers, MA, USA; catalogue number 7074).

### 4.4. Biotin Pull-Down

Biotin pull-downs were carried out according to previous reports [22,25,26,30] with slight modifications. Cells were homogenized in 2.5 mL of lysis buffer, supplemented with 50 mM of *N*-ethylmaleimide (Sigma, St. Louis, MO, USA) and a complete protease inhibitor cocktail (Roche Applied Science, Penzberg, Germany). Lysates were then passed through a 20G needle 10 times and centrifuged for 5 min at 14,000× *g* at 4 °C. The supernatants were applied to a PD10 desalting column (GE Healthcare, Chicago, IL, USA), previously equilibrated with 25 mL of binding buffer supplemented with 50 mM of *N*-ethylmaleimide. Eluates were incubated with 150 μL of NeutrAvidin agarose beads suspension (Thermo Fisher Scientific, Waltham, MA, USA) and gentle rolling for 40 min at room temperature and 2 h and 20 min at 4 °C. Beads were then washed twice with washing buffer (WB) 1, thrice with WB2, once with WB3, thrice with WB4, once again with WB1, once with lysis buffer and thrice with WB5. The ubiquitinated material was eluted from the neutravidin beads with 80 μL of elution buffer by heating the Eppendorf tubes at 95 °C for 5 min. Finally, samples were centrifuged at 16,000× *g* at room temperature for 2 min in a Vivaclear Mini 0.8 μm PES-micro-centrifuge filter unit (Sartorious, Göttingen, Germany) to discard the NeutrAvidin resin.

Buffer compositions were as follows: lysis buffer, 8 M urea, 1% SDS; binding buffer, 3 M urea, 1 M NaCl, 0.25% SDS; WB1, 8 M urea, 0.25 SDS; WB2, 6 M guanidine-HCl; WB3, 6.4 M urea, 1 M NaCl, 0.2% SDS; WB4, 4 M urea, 1 M NaCl, 10% isopropanol, 10% ethanol, 0.2% SDS; WB5, 2% SDS; elution buffer, 250 mM Tris-HCl, pH 7.5, 40% glycerol, 4% SDS, 0.2% bromophenol blue and 100 mM DTT. All buffers, except the elution buffer, were prepared in PBS.

### 4.5. Silver Staining

10% of the neat elution samples were resolved by SDS-PAGE in 4–12% Bolt Bis-Tris Plus pre-cast gels (Invitrogen, Carlsbad, CA, USA). Afterwards, gels were fixed for 1 h at room temperature with 40% methanol and 10% acetic acid containing solution and, then, stained using the SilverQuest silver staining kit (Invitrogen, Carlsbad, CA, USA) according to manufacturer’s instructions.

### 4.6. In-Gel Trypsin Digestion and Peptide Extraction

Eluates from biotin pull-down assays were resolved by SDS-PAGE using 4–12% Bolt Bis-Tris Plus pre-cast gels (Invitrogen, Carlsbad, CA, USA) and visualized with GelCode Blue Stain reagent following manufacturer’s instructions (Thermo Fisher Scientific, Waltham, MA, USA). After the exclusion of avidin monomers and dimer, each lane was cut into four slices and subjected to in-gel digestion as described previously [48]. Briefly, DTT and chloroacetamide were used for protein reduction and alkylation, respectively. Digestion of proteins was performed by incubation of the gel slices with trypsin and overnight incubation at 37 °C. Next day, resulting peptides were extracted from the gel, dried down in a vacuum centrifuge and stored at −20 °C. Peptide mixtures were resuspended in 0.1% of formic acid previous to the LC-MS/MS analysis.

### 4.7. LC-MS/MS Analysis

USP1 mass spectrometric analyses were performed on an EASY-nLC 1000 liquid chromatography system interfaced via a nanospray flex ion source with a Q Exactive (Thermo Fisher Scientific, Waltham, MA, USA) mass spectrometer. Peptides were loaded onto an Acclaim PepMap100 pre-column (75 mm × 2 cm, Thermo Fisher Scientific, Waltham, MA, USA) connected to an Acclaim PepMap RSLC (50 mm × 15 cm, Thermo Fisher Scientific, Waltham, MA, USA) analytical column. Peptides were eluted from the columns using a linear gradient of 2 to 40% acetonitrile in 0.1% of formic acid at a flow rate of 300 nL min^−1^ over 45 min. The mass spectrometers were operated in positive ion mode. Full MS scans were acquired from *m*/*z* 300 to 1850 with a resolution of 70,000 at *m*/*z* 200. The 10 most intense ions were fragmented by high-energy collision dissociation (HCD) with normalized collision energy of 28 and MS/MS spectra were recorded with a resolution of 17,500 at *m*/*z* 200. The maximum injection time was 120 ms for both survey and MS/MS scans, whereas AGC target values of 3 × 10^6^ and 5 × 10^5^ were used for survey and MS/MS scans, respectively. In order to avoid repeat sequencing of peptides, dynamic exclusion was applied for 45 s. Singly charged ions or ions with unassigned charge state were also excluded from MS/MS. Data were acquired using Xcalibur software (Thermo Fisher Scientific, Waltham, MA, USA).

USP9X mass spectrometric analyses were performed on an EASY-nLC 1200 liquid chromatography system interfaced via a nanospray flex ion source with Q Exactive HF-X (Thermo Fisher Scientific, Waltham, MA, USA). Peptides were loaded onto an Acclaim PepMap100 pre-column (75 mm × 2 cm, Thermo Fisher Scientific, Waltham, MA, USA) connected to an Acclaim PepMap RSLC (50 mm × 25 cm, Thermo Fisher Scientific, Waltham, MA, USA) analytical column. Peptides were eluted from the columns using a two-step gradient of 2.4 to 24% (90 min) and 24 to 32% (2 min) acetonitrile in 0.1% of formic acid at a flow rate of 300 nL min^−1^ over 92 min. The mass spectrometers were operated in positive ion mode. Full MS scans were acquired from *m*/*z* 375 to 1850 with a resolution of 60,000 at *m*/*z* 200. The 10 most intense ions were fragmented by high-energy collision dissociation (HCD) with normalized collision energy of 28 and MS/MS spectra were recorded with a resolution of 15,000 at *m*/*z* 200. The maximum injection time was 50 ms for survey and 100 ms for MS/MS scans, whereas AGC target values of 3 × 10^6^ and 1 × 10^5^ were used for survey and MS/MS scans, respectively. In order to avoid repeat sequencing of peptides, dynamic exclusion was applied for 20 s. Singly charged ions or ions with unassigned charge state were also excluded from MS/MS. Data were acquired using Xcalibur software (Thermo Fisher Scientific, Waltham, MA, USA).

USP42 mass spectrometric samples were analyzed using a Orbitrap Fusion Lumos mass spectrometer (Thermo Fisher Scientific, San Jose, CA, USA) coupled to an EASY-nLC 1000 (Thermo Fisher Scientific (Proxeon), Odense, Denmark). Peptides were loaded directly onto the analytical column and were separated by reversed-phase chromatography using a 50-cm column with an inner diameter of 75 μm, packed with 2 μm C18 particles spectrometer (Thermo Fisher Scientific, San Jose, CA, USA). Chromatographic gradients started at 5% acetonitrile, 0.1% formic acid with a flow rate of 300 nL/min for 5 min and and gradually increased to 22% acetonitrile, 0.1% formic acid in 79 min and then to 35% acetonitrile, 0.1% formic acid in 11 min. The mass spectrometer was operated in positive ionization mode with nanospray voltage set at 2.4 kV and source temperature at 275 °C. The acquisition was performed in data-dependent acquisition (DDA) mode and full MS scans with 1 micro scans at resolution of 120,000 were used over a mass range of *m*/*z* 350–1500 with detection in the Orbitrap mass analyzer and auto gain control (AGC) was set to 1E5. The number of selected precursor ions for fragmentation was determined by the “Top Speed” acquisition algorithm and a dynamic exclusion of 60 s. Fragment ion spectra were produced via HCD at normalized collision energy of 28 and they were acquired in the ion trap mass analyzer. AGC was set to 1E4, and an isolation window of 1.6 *m*/*z* and a maximum injection time of 200 ms were used. Data were acquired with Xcalibur software.

LC-MS/MS of USP7 was done by coupling an UltiMate 3000 RSLCnano LC system to a Q Exactive Plus mass spectrometer (Thermo Fisher Scientific, Waltham, MA, USA). Peptides were loaded into a trap column (Acclaim™ PepMap™ 100 C18 LC Columns 5 µm, 20 mm length) for 3 min at a flow rate of 10 µL/min in 0.1% formic acid. Then, peptides were transferred to an EASY-Spray PepMap RSLC C18 column (Thermo Fisher Scientific, Waltham, MA, USA) (2 µm, 75 µm × 50 cm) operated at 45 °C and separated using a 90 min gradient (buffer A: 0.1% FA; buffer B: 100% ACN, 0.1% FA) at a flow rate of 250 nL/min. The gradient used was from 4 to 6% B in 2.5 min, from 6 to 25% B in 72 min, from 25 to 42.5% B in 15.5 min plus 6.5 additional minutes at 98% B. Peptides were sprayed at 1.8 kV into the mass spectrometer via the EASY-Spray source. The capillary temperature was set to 300 °C. The mass spectrometer was operated in a data-dependent mode, with an automatic switch between MS and MS/MS scans using a top 15 method. Intensity threshold ≥ 6.7 × 10^4^, dynamic exclusion of 40 s and excluding charges +1 and > +6. MS spectra were acquired from 350 to 1500 *m*/*z* with a resolution of 70,000 FWHM (200 *m*/*z*). Ion peptides were isolated using a 2.0 Th window and fragmented using HCD with a normalized collision energy of 27. MS/MS spectra resolution was set to 17,500 (200 *m*/*z*). The ion target values were 3e6 for MS (maximum IT of 25 ms) and 1e5 for MS/MS (maximum IT of 45 ms).

For USP11, each sample was subjected to 2D-nano LC ESI-MSMS analysis using a nano liquid chromatography system (Eksigent Technologies nanoLC Ultra 1D plus, AB SCIEX, Foster City, CA, USA) coupled to high speed Triple TOF 5600 mass spectrometer (SCIEX, Foster City, CA, USA) equipped with a Nanospray III source. Injection volume was 5 µL. The analytical column used was a silica-based reversed phase column nanoACQUITY UPLC 75 µm × 15 cm (Waters, Milford, MA, USA, Waltham, MA, USA), 1.7 µm particle size. The trap column was an Acclaim PepMap 100 (Thermo Fisher Scientific, Waltham, MA, USA), 5 µm particle diameter, 100 Å pore size, switched on-line with the analytical column. The loading pump delivered a solution of 0.1% formic acid in water at 2 µL/min. The nano-pump provided a flow-rate of 250 nL/min and was operated under gradient elution conditions, using 0.1% formic acid in water as mobile phase A, and 0.1% formic acid in acetonitrile as mobile phase B. Gradient elution was performed according to the following scheme: isocratic conditions of 96% A: 4% B for five minutes, a linear increase to 40% B in 60 min, then a linear increase to 90% B for 5 additional minutes, isocratic conditions of 90% B for ten minutes and return to initial conditions in 2 min. Total gradient length was 100 min. Data acquisition was performed with a TripleTOF 5600 System. Ionization occurred under the following conditions: ionspray voltage floating (ISVF) 2800 V, curtain gas (CUR) 20, interface heater temperature (IHT) 150 °C, ion source gas 1 (GS1) 20, declustering potential (DP) 85 V. All data was acquired using information-dependent acquisition (IDA) mode with Analyst TF 1.7 software (AB SCIEX, Foster City, CA, USA). For IDA parameters, 0.25 s MS survey scan in the mass range of 350–1250 Da were followed by 15 MS/MS scans of 150 ms in the mass range of 100–1500 (total cycle time: 1.8 s). Switching criteria were set to ions greater than mass to charge ratio (*m*/*z*) 350 and smaller than *m*/*z* 1250 with charge state of 2–5 and an abundance threshold of more than 90 counts (cps). Former target ions were excluded for 20 s. IDA rolling collision energy (CE) parameters script was used for automatically controlling the CE.

The mass spectrometry proteomics data have been deposited to the ProteomeXchange Consortium via the PRIDE [49] partner repository with the dataset identifier PXD025443.

### 4.8. Data Processing and Bioinformatics Analysis

Acquired raw data files were processed with the MaxQuant [50] software (versions 1.5.3.17 and 1.6.0.16) using the internal search engine Andromeda [51] and searched against the UniProtKB database restricted to Homo sapiens (20,187 entries). Spectra originated from the different slices corresponding to the same biological sample were combined. Carbamidomethylation (C) was set as fixed modification, whereas Met oxidation, protein *N*-terminal acetylation and Lys GlyGly (not *C*-term) were defined as variable modifications. Mass tolerance was set to 8 and 20 ppm at the MS and MS/MS level, respectively; except in the analysis of the TOF data for which the values of 0.006 Da and 40 ppm were used, respectively. Enzyme specificity was set to trypsin, allowing for cleavage *N*-terminal to Pro and between Asp and Pro with a maximum of two missed cleavages. Match between runs option was enabled with 1.5 min match time window and 20 min alignment window to match identification across samples. The minimum peptide length was set to seven amino acids. The false discovery rate for peptides and proteins was set to 1%. Normalized spectral protein label-free quantification (LFQ) intensities were calculated using the MaxLFQ algorithm [52].

### 4.9. Data Analysis

MaxQuant output data was analysed with Perseus (version 1.6.0.7) [53]. First, proteins only identified by site, contaminants, reverse hits and proteins with no unique peptides and/or no intensity were removed. Then, missing LFQ intensity values were replaced with values from a normal distribution (width 0.3 and down shift 1.8), meant to simulate expression levels below the detection limit (45). Statistically significant differences in protein abundance were determined by two-tailed Student’s *t*-test. Putative DUB substrates should fulfil the following criteria: (1) display a LFQ fold change (siRNA vs control) above 2 that is statistically significant (*p* < 0.05); (2) be detected by at least two unique peptides; and (3) contain no imputed values in any of the three replicas of at least one of the conditions, or have a maximum of one imputed value in each condition.

In the case of Gly Gly peptides, reverse hits, contaminants, peptides with no intensity and those with a probability below 0.75, given by MaxQuant softwarte, were removed.

### 4.10. Web Application

Resulting files after the statistical analysis were imported into a MySQL database without further filtering in order to allow the users to choose their own filtering criteria using the web interface. The schema of this database is available in Figure S7, being Experiment and Evidence the central tables. A web application was developed using Java EE technology to display and query this database. The source code is available at: https://github.com/akrogp/EhuBio/tree/master/Projects/java/Dubase/java, accessed date 30 April 2021. All the original data files used for feeding the database are available to the user for download from the experiment details page shown within the web application.

## Figures and Tables

**Table 1:** Putative deubiquitinating enzyme (DUB) substrates.

Silenced DUB	Gene Name	Description	FC ^1^
USP1	BCOR	BCL-6 corepressor	2.43
	PCNA	Proliferating cell nuclear antigen	1.49
	GLUL	Glutamine synthetase	1.12
USP7	TTK	Dual specificity protein kinase TTK	2.33
	GABPA	GA-binding protein alpha chain	1.66
	RPL29	60S ribosomal protein L29	1.61
	RNF2	E3 ubiquitin-protein ligase RING2	1.54
	CREB1	Cyclic AMP-responsive element-binding protein 1	1.44
	MARCKS	Myristoylated alanine-rich C-kinase substrate	1.21
USP9X	RNF10	RING finger protein 10	3.94
	RPS25	40S ribosomal protein S25	3.59
	CCND2	G1/S-specific cyclin-D2	3.32
	TMEM259	Membralin	3.00
	AAMP	Angio-associated migratory cell protein	2.77
	VHL	Von Hippel-Lindau disease tumor suppressor	2.18
	SEPT6	Septin-6	2.17
	MICALL1	MICAL-like protein 1	2.14
	CCDC124	Coiled-coil domain-containing protein 124	1.95
	CORO7	Coronin-7	1.91
	DLG3	Disks large homolog 3	1.82
	RPL13A	60S ribosomal protein L13a	1.71
	TCP11L2	T-complex protein 11-like protein 2	1.56
	GPRASP2	G-protein coupled receptor-associated sorting protein 2	1.25
	ARAF	Serine/threonine-protein kinase A-Raf	1.20
	GNA12	Guanine nucleotide-binding protein subunit alpha-12	1.19
	ZNF598	Zinc finger protein 598	1.19
	ZC3H15	Zinc finger CCCH domain-containing protein 15	1.08
	CEP170	Centrosomal protein of 170 kDa	1.06
	PDIA4	Protein disulfide-isomerase A4	1.00
USP11	RPS2	40S ribosomal protein S2	4.17
	UBE2L3	Ubiquitin-conjugating enzyme E2 L3	2.15
	RPS7	40S ribosomal protein S7	1.33
USP42	SLC20A1	Sodium-dependent phosphate transporter 1	4.09
	WARS	Tryptophan--tRNA ligase, cytoplasmic	3.86
	CUL2	Cullin-2	2.94
	UBR7	Putative E3 ubiquitin-protein ligase UBR7	2.86
	CPD	Carboxypeptidase D	2.31
	ARHGAP17	Rho GTPase-activating protein 17	2.30
	SETD3	Histone-lysine *N*-methyltransferase setd3	2.10
	GTF2H1	General transcription factor IIH subunit 1	2.04
	CORO1C	Coronin-1C	1.95
	HMOX1	Heme oxygenase 1	1.69
	PRKAR2A	cAMP-dependent protein kinase type II-alpha regulatory subunit	1.65
	DNAJC21	DnaJ homolog subfamily C member 21	1.52
	FUBP3	Far upstream element-binding protein 3	1.40
	PES1	Pescadillo homolog	1.38
	SMARCD2	SWI/SNF-related matrix-associated actin-dependent regulator of chromatin subfamily D member 2	1.36
	LSR	Lipolysis-stimulated lipoprotein receptor	1.26
	IFT81	Intraflagellar transport protein 81 homolog	1.25
	RAD50	DNA repair protein RAD50	1.23
	RNF2	E3 ubiquitin-protein ligase RING2	1.11
	PJA2	E3 ubiquitin-protein ligase Praja-2	1.10
	PSMC6	26S protease regulatory subunit 10B	1.07
	BUB3	Mitotic checkpoint protein BUB3	1.06
	BTBD2	BTB/POZ domain-containing protein 2	1.05
	FABP5	Fatty acid-binding protein, epidermal	1.02

^1^ FC: siRNAi/Control Fold Change in log_2_ scale.

## Data Availability

All mass spectrometry raw data are available via ProteomeXchange with identifier PXD025443.

## Supplementary Materials

The following are available online at https://www.mdpi.com/article/10.3390/ijms22094851/s1.
