# Supplementary material for: A Proteomic Approach for Systematic Mapping of Substrates of Human Deubiquitinating Enzymes
Source: Int J Mol Sci. 2021 May 3;22(9):4851. doi: 10.3390/ijms22094851 (PMC8124392; doi:10.3390/ijms22094851)
Supplement: Supplementary file 1 [file ijms-22-04851-s001.zip › Supplementary Figures.pdf]

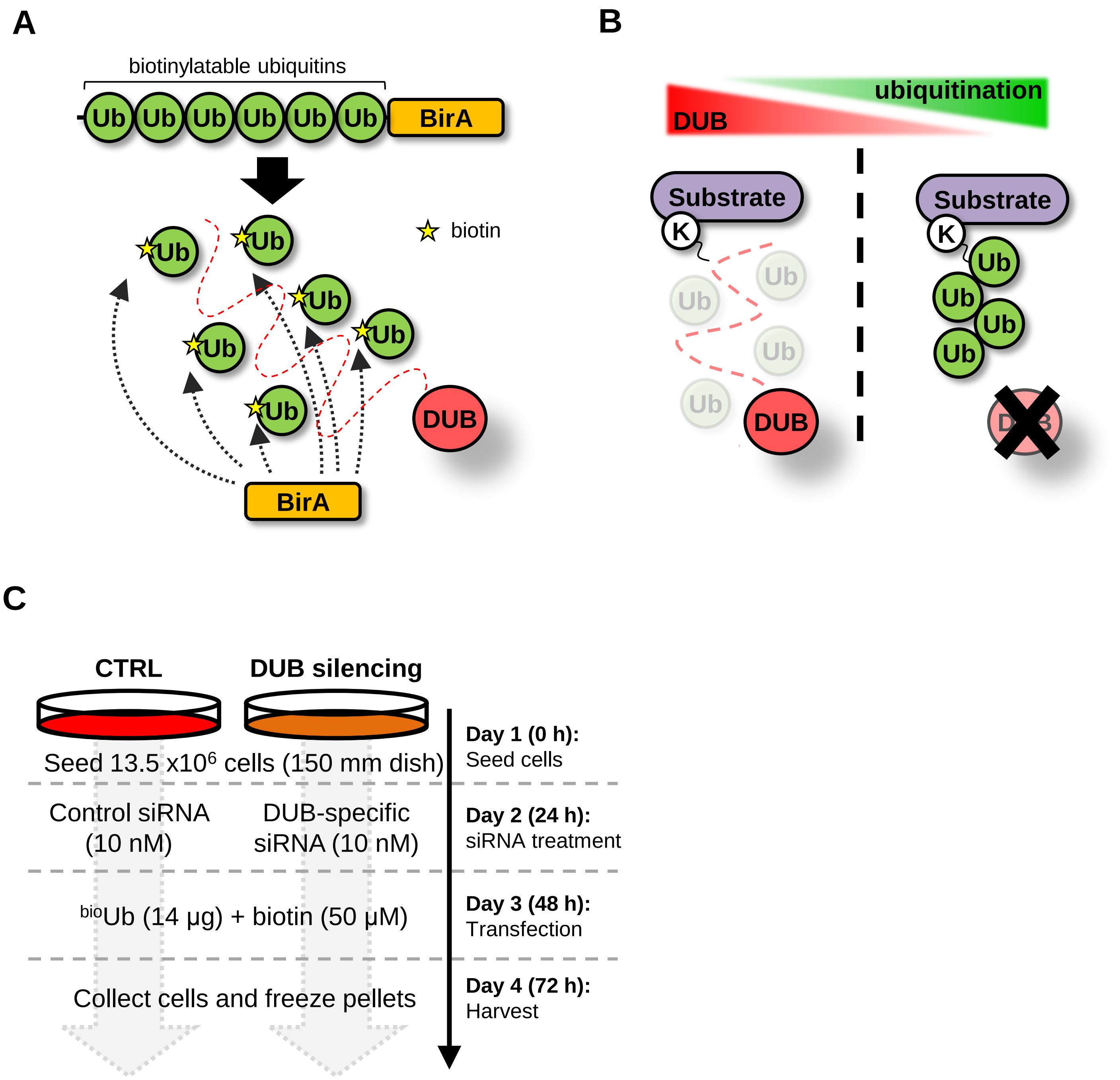

Figure S1. Workflow for the identification of putative DUB substrates. A. The ^{bio}Ub strategy. A plasmid composed of six ubiquitin, each of them modified at their N-terminus with a minimal biotinylatable peptide, and the *E. coli* BirA enzyme is expressed in the cells. This construct is synthesized as a precursor polypeptide that needs to be digested by endogenous DUBs, so ubiquitins and BirA are released. Free BirA is then able to conjugate biotin (depicted with a yellow star) to each ubiquitin, which are used by the endogenous machinery to ubiquitinate cellular proteins. Proteins modified with the biotinylated ubiquitin can be easily purified due to the strength of biotin-avidin interaction. **B.** Working hypothesis. Given that DUBs are in charge of removing ubiquitin from substrates, the silencing of one DUB should render a higher ubiquitination of its substrates. **C.** Experimental workflow. 24 h after seeding, HEK293 cells were transfected with DUB-specific siRNAs, to ensure a reduction of DUB levels before the biotinylated ubiquitin is available to the cellular machinery. A siRNA with no significant sequence similarity to mouse, rat or human genes was used as control. siRNAs were used at a concentration of 10 nM. Next day, cells were transfected with the ^{bio}Ub plasmid and supplemented with 50 µM of biotin. Cells pellets were collected and frozen 24 h later. Purification procedure of proteins modified by biotinylated ubiquitin is based on avidin-biotin strong interaction, which allows stringent washes of the beads.

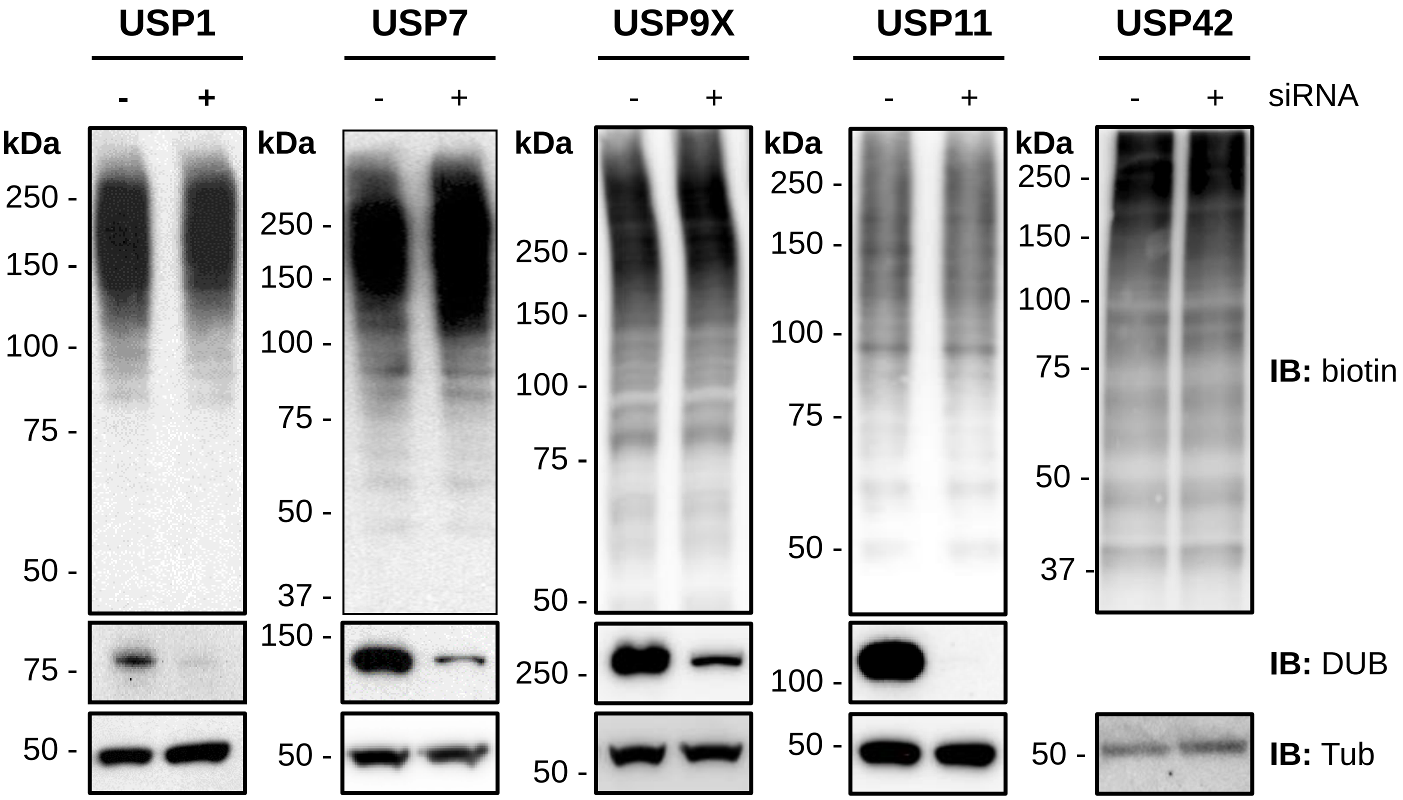

Figure S2. Levels of conjutaged ubiquitin after DUB silencing. Biotin immunoblots on cell whole extracts showed that the levels of conjugated ubiquitin are not globally altered after DUB silencing. DUB levels were detected with specific antibodies to each DUB, except for USP42. Anti-Tubulin antibody was used as loading control.

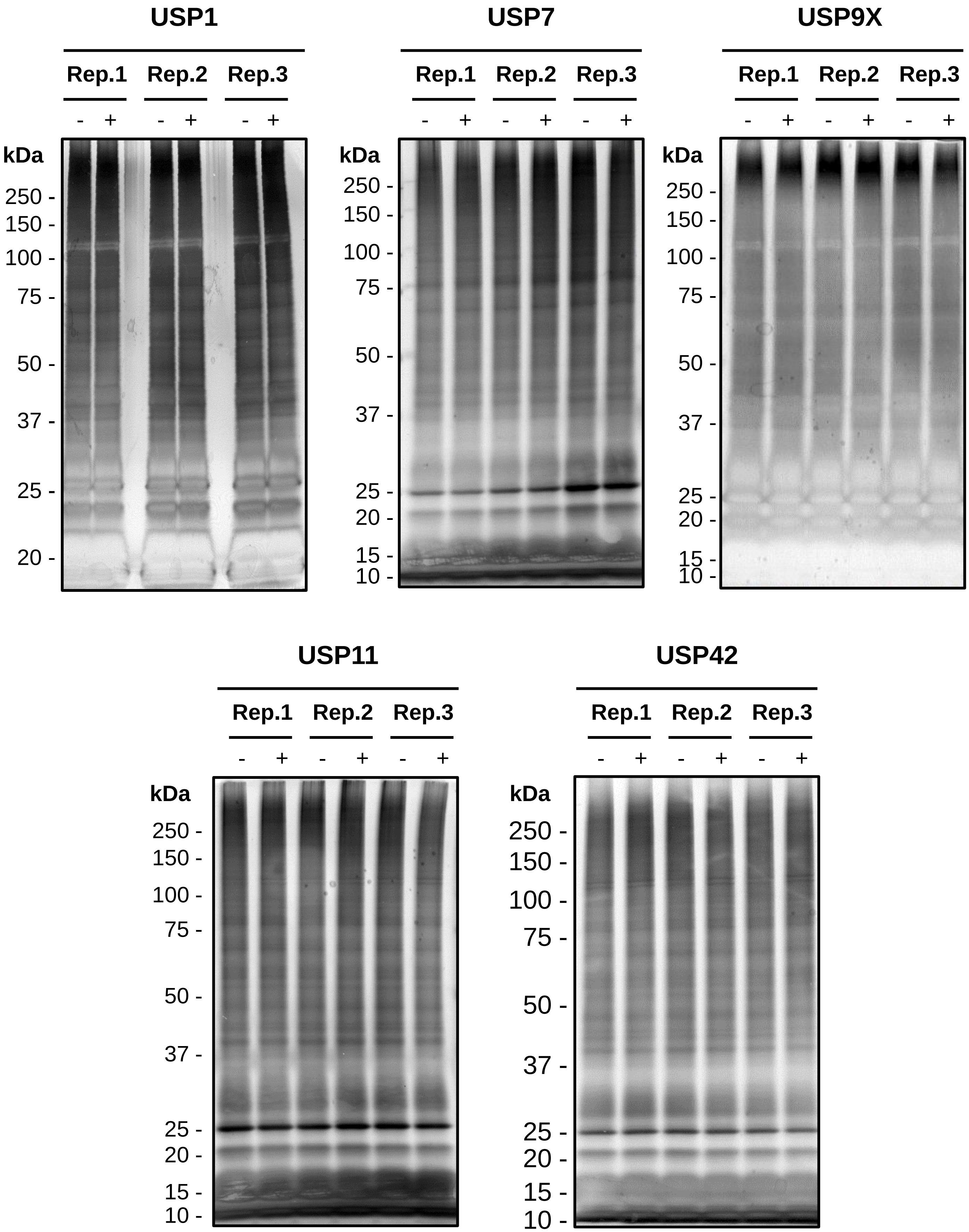

Figure S3. Amount of ubiquitinated proteins isolated by biotin pull-downs from control and DUB-silenced samples. Levels of isolated proteins, detected by silver staining, were equivalent in control (-) and siRNA-treated samples (+).

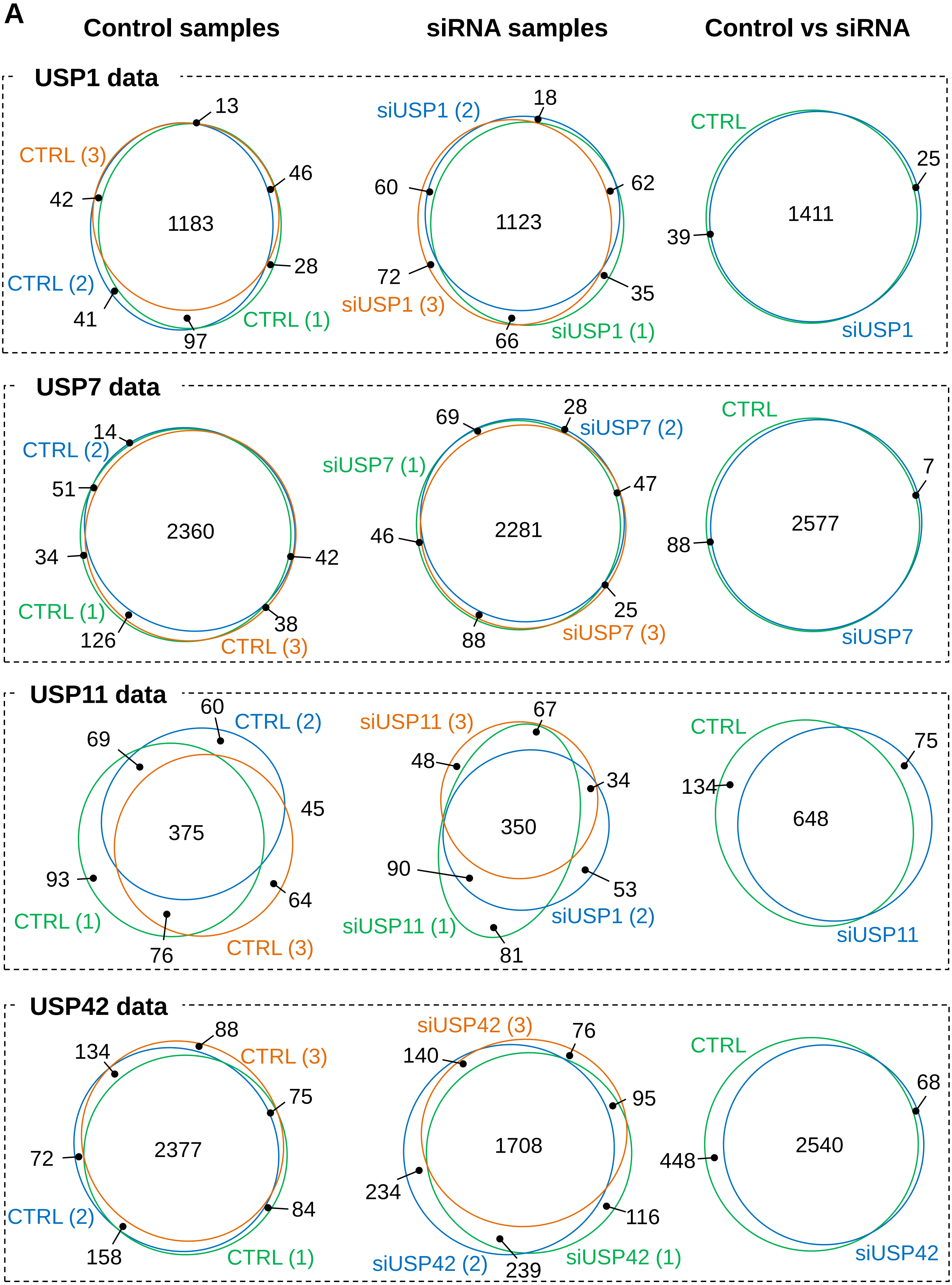
A
Control samples
siRNA samples
Control vs siRNA
USP1 data
CTRL (3)
CTRL (2)
CTRL (1)
13
46
42
1183
28
41
97
siUSP1 (2)
siUSP1 (3)
siUSP1 (1)
18
60
62
1123
72
35
66
CTRL
siUSP1
25
1411
39
USP7 data
CTRL (2)
CTRL (1)
CTRL (3)
14
51
2360
34
42
126
38
siUSP7 (1)
siUSP7 (2)
siUSP7 (3)
69
28
47
2281
46
25
88
CTRL
siUSP7
7
2577
88
USP11 data
CTRL (2)
CTRL (1)
CTRL (3)
60
69
45
375
93
64
76
siUSP11 (3)
siUSP11 (1)
siUSP1 (2)
67
48
34
350
90
53
81
CTRL
siUSP11
75
134
648
USP42 data
CTRL (3)
CTRL (2)
CTRL (1)
88
134
75
2377
72
84
158
siUSP42 (3)
siUSP42 (2)
siUSP42 (1)
76
140
95
1708
234
116
239
CTRL
siUSP42
68
2540
448

Figure S4

B

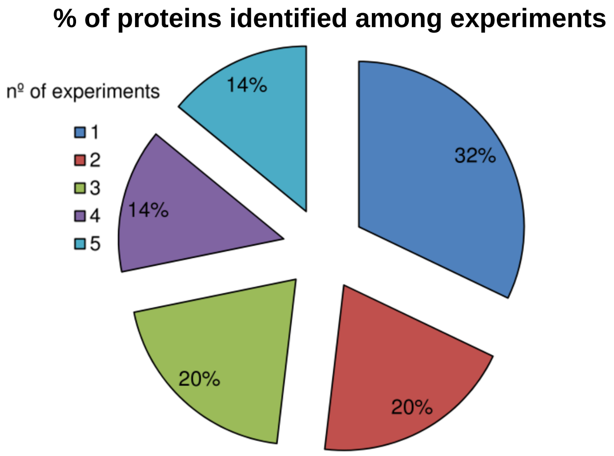

C

	USP1	USP7	USP9X	USP11	USP42
**USP1**	100 %	88 %	95 %	86 %	91 %
**USP7**		100 %	91 %	97 %	80 %
**USP9X**			100 %	97 %	89 %
**USP11**				100 %	96 %
**USP42**					100 %

D

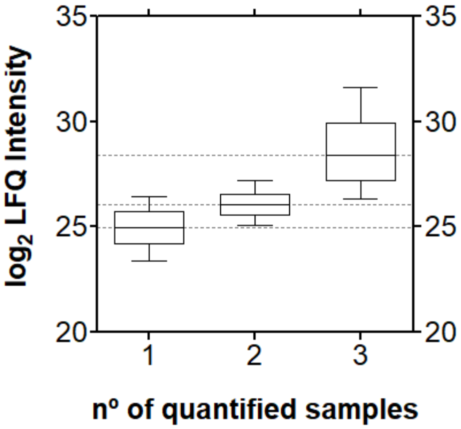

E

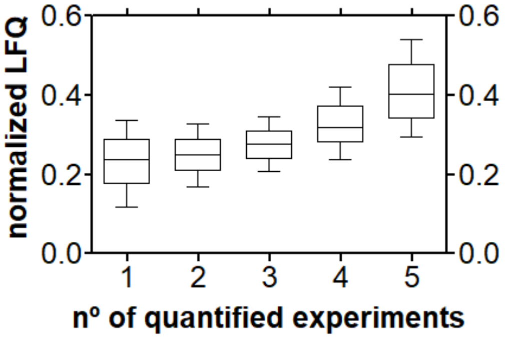

Figure S4. Reproducibility of MS analysis. A. Overlap between proteins identified among the three replica of each condition, as well as between both conditions, is shown. Typically, an 80 % overlap across replicas of the same condition and an 80-95 % overlap between control and siRNA conditions were observed. **B.** Percentage of proteins identified across the control samples of the five experiments. About 70 % of the total proteins identified were detected in at least two out of five experiments. **C.** Overlap between proteins identified among the controls of the five experiments. The overlap between two experiments is referred to as the percentage of proteins from the smaller dataset found in the bigger dataset. **D.** Abundance of the proteins identified across the three USP9X control replicas. Abundance of individual protein was determined by their LFQ intensities (displayed in log_2 scale). Proteins consistently quantified across the three replicas were those with a higher abundance. **E.** Abundance of the proteins identified across the controls of the five experiments. Proteins consistently quantified across the control samples of the different experiment were those presenting a higher abundance. Abundance is determined by their LFQ Intensity values, which were normalized to a 0 to 1 range.

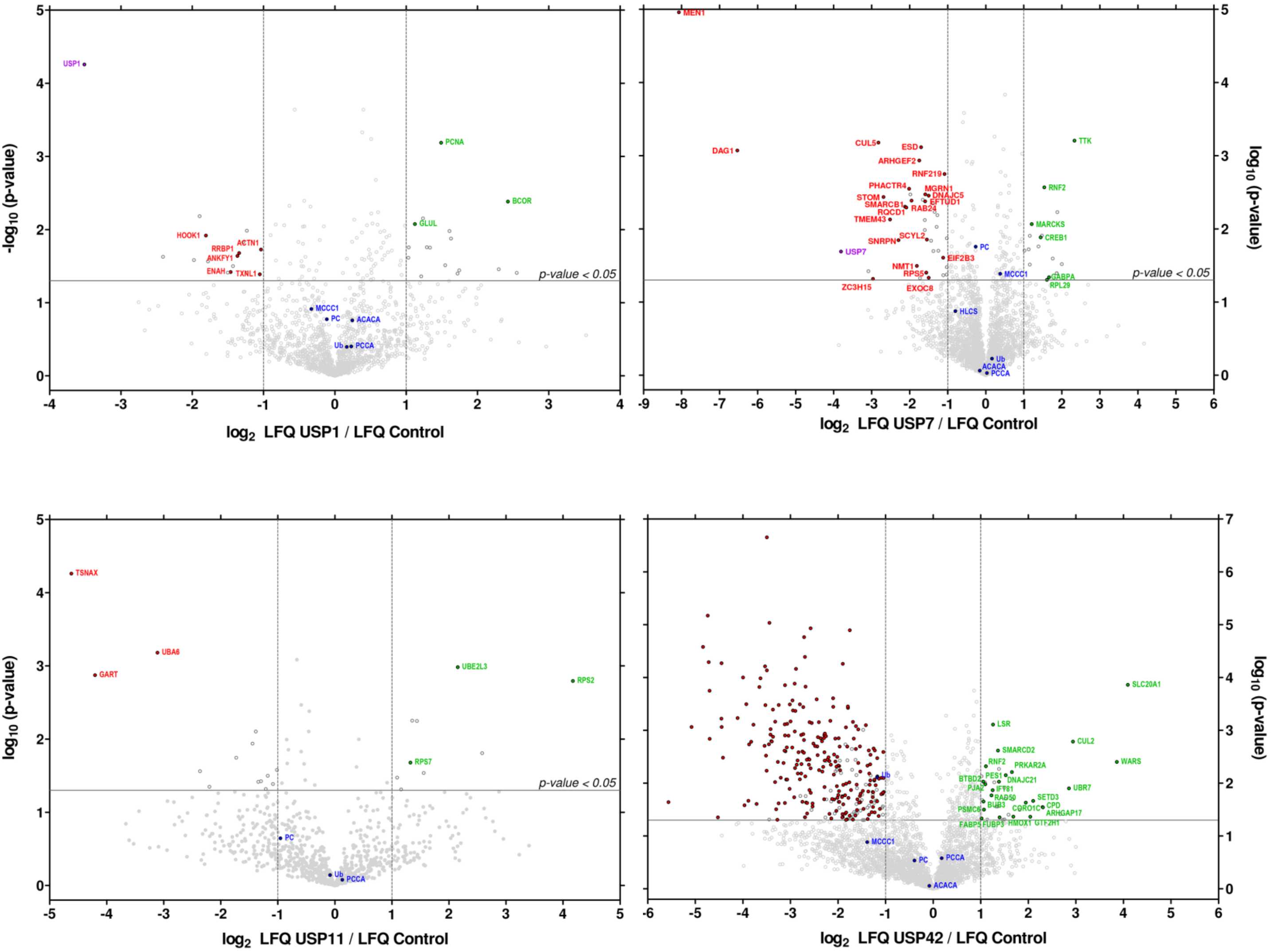

Figure S5. Putative DUB substrates of the USP1, USP7, USP11 and USP42 DUBs. Volcano plot shows differentially ubiquitinated proteins upon DUB silencing, relative to control samples. Abundance of each individual protein was determined by their LFQ intensities. The LFQ siRNA/control ratios ($\log_2$ scale) and the *t*-test p-values (–log10 scale) are displayed in the *X* and *Y* axis, respectively. The statistical significance (p-value < 0.05) is indicated with a horizontal grey line, while vertical grey lines depict a 2-fold increase or decrease of the ubiquitinated levels upon DUB silencing. Proteins with a 2-fold increase or reduction of their ubiquitination levels upon DUB silencing are shown in green and red respectively. The silenced DUBs are shown in purple. Endogenously ubiquitinated proteins and ubiquitin are coloured in blue.

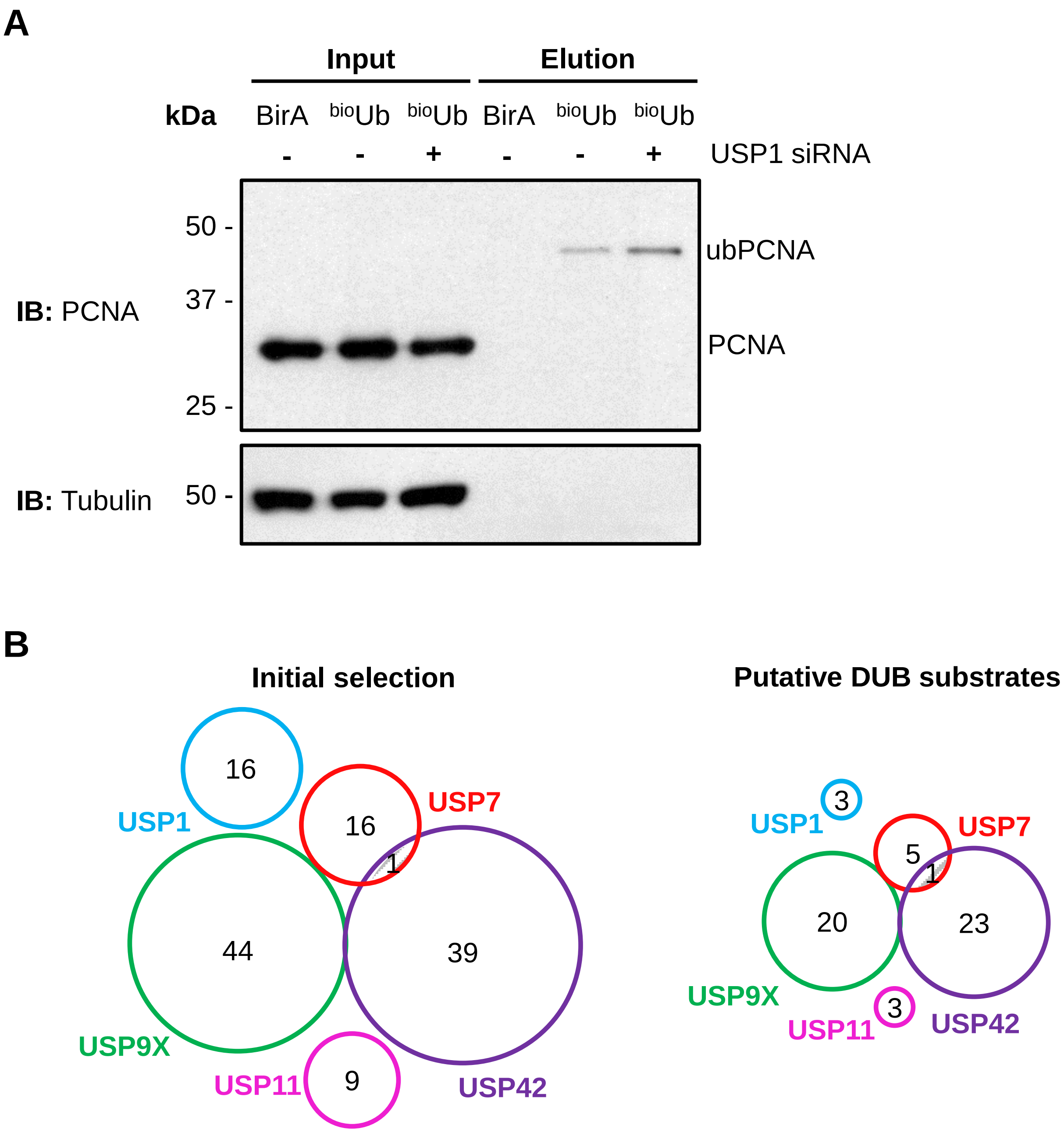

Figure S6. PCNA validation and overlap of putative DUB substrates. A. Immunoblot with anti-PCNA-antibody confirmed the increase ubiquitination detected by MS in siUSP1-silenced samples, compared to controls. Whole cell extracts (input) and ubiquitin-enriched material (elution) from biotin pulldown were analysed by Western blot. Mono-ubiquitinated PCNA was detected from the samples where the biotinylated ubiquitin is expressed (^{bio}Ub), which relative to the non-modified PCNA (inputs) showed an increase of approximately 10 kDa in their molecular weight. An increase ubiquitination of PCNA is observed when siUSP1 is silenced (+), relative to scramble siRNA (-). As control, samples that expressed only the BirA enzyme were used. **B.** Overlap of putative substrates of the studied DUBs. Venn diagrams display the overlap between proteins found more ubiquitinated in each analysis (LFQ siRNA/control ratio > 2, p-value 0.05) and the overlap between those considered confident putative DUB substrates.

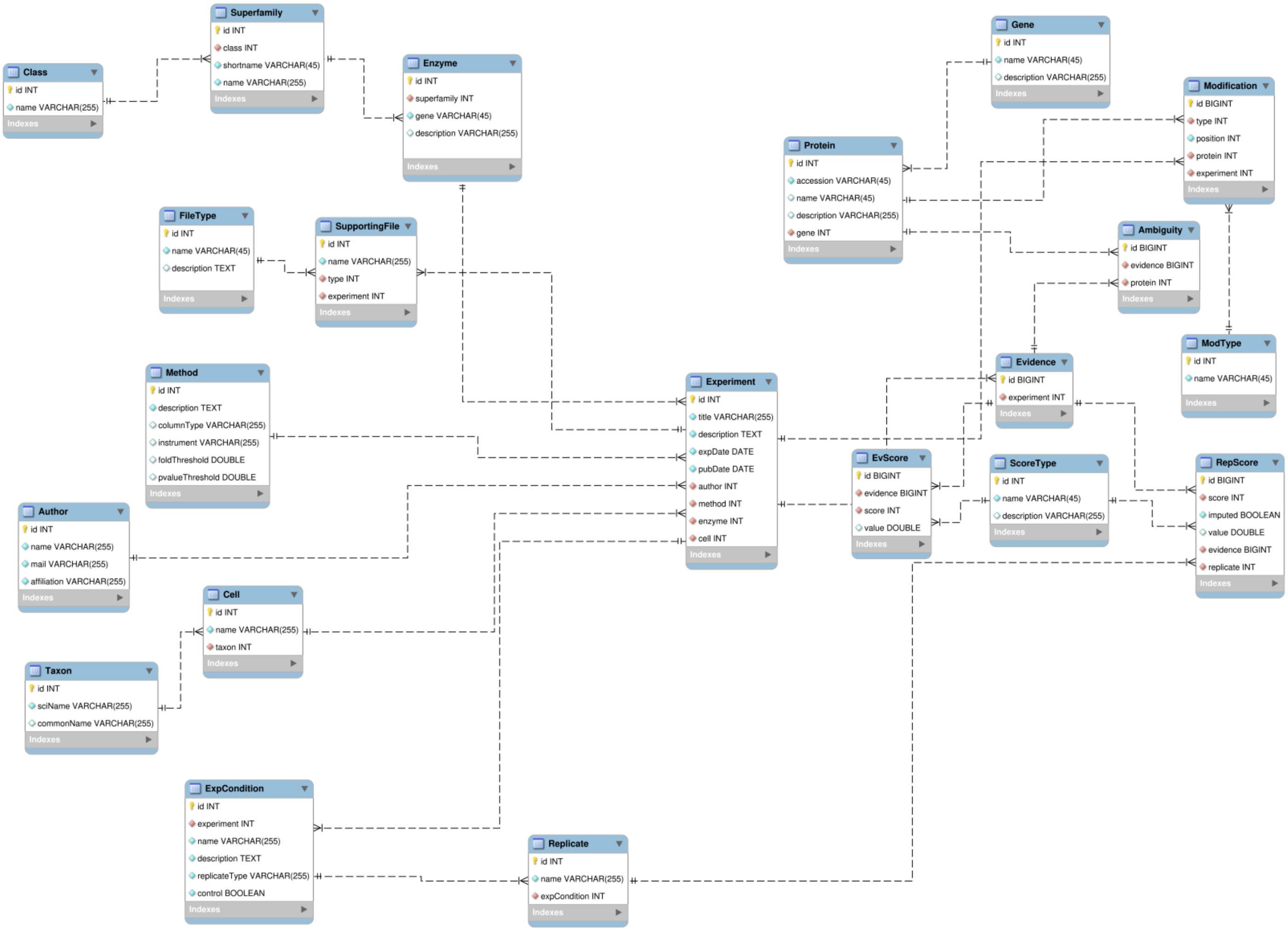

Figure S7. MySQL database schema for DUBase. Experiment and Evidence are the key tables that lead most of the queries. The Experiment table organises all the information relative to an experiment, which it is distributed in different secondary tables in order to avoid redundancies. The Evidence table is used to relate all the evidences (a term we use for the results or identifications reported) to a given experiment. This table is referenced itself by other secondary tables that hold specific results for this specific evidence such as a global score, the scores of the different replicates, the protein ambiguity group members and the post-translational protein modifications. All the tables have a numeric primary key (called id) for efficiency purposes.

Table S1. Proteins identified in LC-MS/MS analysis. Putative DUB substrates are highlighted in green, while those found significantly less ubiquitinated upon DUB silencing are shown in red.

Table S2. GlyGly sites identified in LC-MS/MS analysis. Only those with a probability above 0,75, given by MaxQuant software, are provided.
